# Role of Inflammatory Risk Factors in the Pathogenesis of *Streptococcus pneumoniae*

**DOI:** 10.3389/fimmu.2018.02275

**Published:** 2018-10-02

**Authors:** Ifrah Sohail, Sumit Ghosh, Santhosh Mukundan, Susan Zelewski, M. Nadeem Khan

**Affiliations:** Biomedical Sciences, University of North Dakota, Grand Forks, ND, United States

**Keywords:** carriage, inflammation, pneumococcus, pathogenesis, co-infection

## Abstract

*Streptococcus pneumoniae* (*Spn*) is a colonizer of the human nasopharynx (NP), causing a variety of infections in humans including otitis media, pneumonia, sepsis, and meningitis. The NP is an immune permissive site which allows for the persistence of commensal bacteria. Acute or chronic respiratory airway inflammation constitutes a significant risk factor for the manifestation of *Spn* infections. The inflammatory conditions caused by an upper respiratory viral infection or respiratory conditions such as allergic asthma and chronic obstructive pulmonary disorders (COPDs) are implicated in the dysregulation of airway inflammation and tissue damage, which compromise the respiratory barrier integrity. These immune events promote bacterial outgrowth leading to *Spn* dissemination and invasion into the bloodstream. Therefore, suppression of inflammation and restoration of respiratory barrier integrity could contain *Spn* infections manifesting in the backdrop of an inflammatory disease condition. The gained knowledge could be harnessed in the design of novel therapeutic interventions to circumvent *Spn* bacterial infections.

## *Spn* carriage, diseases, and vaccines

*Spn* diseases constitute a major global health problem (1). It is estimated that about one million US adults contract *Spn* pneumonia each year, which accounts for 5–7% of annual mortality (2), and the mortality rate for invasive *Spn* diseases (IPDs; sepsis and meningitis) is even higher (3). The vulnerable human populations who are at the highest risk of developing *Spn* infections include infants, the elderly, and immunocompromised patients (4, 5); thus, these populations have a much higher incidence of IPDs (6). Currently available pneumococcal conjugate vaccines (PCVs) target the bacterial capsule antigens and have contributed to a global reduction in the *Spn* disease burden (7). However, the replacement of capsular serotypes has occurred (8, 9), thus requiring an expansion of the valency of current PCV formulations. Therefore, despite continued vaccination programs, *Spn* infections continue to occur and account for significant clinical and economic burden.

*Spn* establishes asymptomatic carriage in the NP of about 20–40% of healthy adults, with even higher frequency in infant and elderly populations (10). Since carriage is a prerequisite for *Spn* infections and diseases (11, 12), at-risk populations are more frequently and persistently colonized by *Spn* (13, 14). The transition of carriage into disease depends on multiple risk factors such as age, inflammatory conditions, geographical area, socio-economic conditions, genetics, and immune system. Additionally, carriage is associated with bacterial dissemination, which leads to the widespread acquisition of *Spn* bacteria in the community (14). However, carriage serves as a double-edged sword: while it constitutes an indispensable state for *Spn* infections, carriage also activates innate and adaptive immunity in the NP, leading to the generation of a *Spn* antigen-specific protective immune response against colonizing *Spn* serotypes (15–18). In mouse models, *Spn* carriage has been shown to trigger a mild inflammatory event resulting in the activation of Toll-Like Receptor-2 (TLR2) and inflammasome (16, 19). Additionally, *Spn* carriage develops antigen-specific antibody and T-cell responses in mouse and human experimental carriage models (20).

Currently available *Spn* conjugate vaccines protect against invasive diseases by preventing the acquisition of *Spn* bacterial carriage of the vaccine serotypes (21). PCVs induce anti-capsular opsonic antibodies which lead to the elimination of *Spn* carriage of vaccine serotypes in the NP (12, 22). However, the elimination of vaccine serotypes has led to the emergence of non-vaccine colonizing and disease-causing replacement serotypes (9, 23). Protein-based *Spn* vaccines are envisioned to protect against *Spn* infections in a serotype-independent manner (24, 25). However, protein-based *Spn* vaccines may or may not eliminate carriage from the NP. Some protein-based *Spn* vaccines aim to maintain low-density stable carriage instead of eliminating it (22–24). It is expected that the maintenance of low-density carriage will limit bacterial virulence evolution, and, consequently, could contain the emergence of new capsule serotypes (24). However, advanced infection models and a substantial amount of data are required to develop this concept further and understand the effect of carriage persistence in the at-risk populations. It should be noted that carriage persistence and its beneficial aspects may differ from one *Spn* serotype to another, since *Spn* has a vast serotype repertoire with diverse colonization kinetics in humans and mouse models, and some invasive serotypes are less effective colonizers (26, 27). Therefore, further studies are required to establish the duration of stable asymptomatic carriage specific to each serotype. Given the nature of *Spn* as a bridge between a commensal and a pathogen, these factors are essential to consider for the development of advanced vaccination or therapeutic strategies that favor the retention of low-density *Spn* carriage in the NP. The answers to these questions necessitate the development of advanced animal infection models that mimic the natural *Spn* infections.

## Respiratory inflammation and *Spn* infections

**Figure 1:**
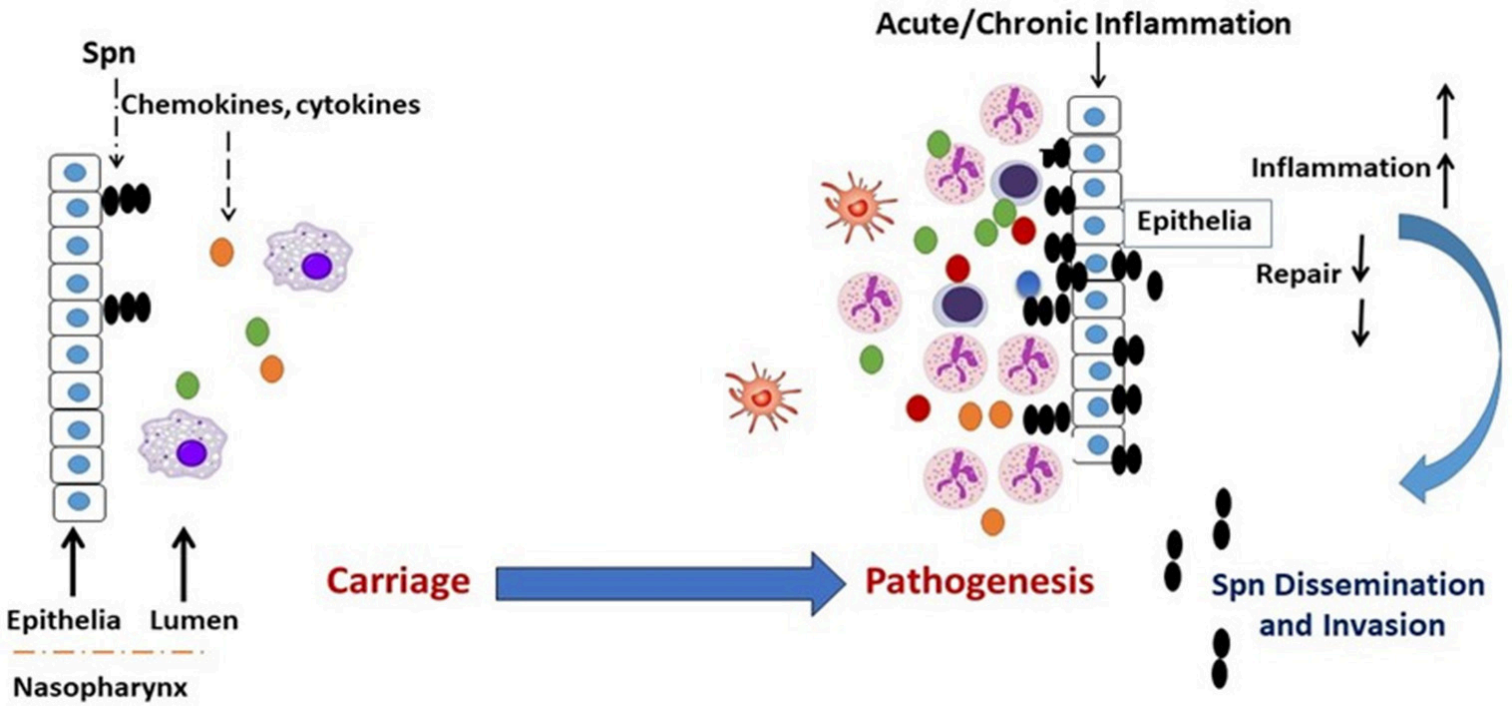
Transition of *Spn* commensal colonization to disease. Acute or chronic airway inflammation caused by influenza virus or chronic conditions such as COPD or allergic asthma promote *Spn* bacterial outgrowth and compromises respiratory tissue barrier function, which leads to the invasion of *Spn* bacteria into the bloodstream establishing invasive infection. Colored dots (green, yellow, red, blue) represent chemokines and cytokines.

Respiratory conditions involving dysregulated and damaging airway inflammation constitute a significant risk factor for the commencement of *Spn* bacterial infections (28–34). While baseline inflammation is required for the gradual clearance of carriage in the NP, hyper and dysregulated inflammation is implicated in epithelial inflammation and tissue damage, which compromises airway barrier integrity and promotes *Spn* outgrowth and dissemination to sterile tissues (34–36). Therefore, a carefully orchestrated inflammatory response is required for the resolution of airway bacterial infections (37, 38), and defects in the regulation of inflammation that arise from respiratory conditions become a contributor to tissue pathology, thus leading to an increased permissiveness for *Spn* infections (Figure 1).

### Influenza virus-induced airway inflammation and *Spn* infections

Airway co-infection with a respiratory virus constitutes the most significant risk factor for *Spn* i**nfections** (36, 39). Clinically, over 50% of young children (<2 years of age) are colonized by *Spn* and other otopathogens in the NP, and there is a significant correlation between the development of bacterial acute otitis media (AOM) and pneumonia with viral upper respiratory co-infections (40–42). A prior influenza viral infection in the NP/lung promotes secondary *Spn* bacterial infections. Similarly, a concurrent viral infection in *Spn-*colonized hosts results in the transition of commensal low-density *Spn* carriage into a *Spn* infection. Traditionally, a majority of murine influenza-*Spn* co-infection models introduced *Spn* in influenza-infected hosts, allowing *Spn* to utilize influenza-mediated airway changes to manifest the disease (43– 46). This model system could be used to study immune mechanisms implicated in the development of post-influenza secondary *Spn* infections. However, since a significant proportion of humans is colonized by *Spn* and other otopathogens in the NP, the introduction of influenza in *Spn*-colonized mice also mimics the natural *Spn* infections (47). The latter infection condition is more appropriate to study the transition from commensal colonizer to disease-causing *Spn* during an influenza viral co-infection (Figure 1). Therefore, both of the aforementioned experimental models should be used to investigate the role of influenza virus in the manifestation of *Spn* infections.

Airway co-infection is characterized by complex interactions between co-infecting pathogens and the host, leading to the disruption of physical barriers, dysregulation of immune responses, and delays in a return to homeostasis (48, 49). Respiratory viruses such as influenza, RSV, parainfluenza, and adenovirus promote *Spn* diseases in the setting of a co-infection (40, 50–54). Influenza virus replication in alveolar epithelia promotes the recruitment of CCR2^hi^ inflammatory monocytes (IMs) and upregulates receptors for bacterial adherence (55–57). IMs act as a double-edged sword; they are required for viral resolution but are implicated in first-line pulmonary epithelial and tissue damage, leading to viral spread (56, 58). CCR2-deficient mice (CCR2^−^/^−^) lacking monocytes and monocyte-derived cells significantly limit lung tissue damage (59). Additionally, CCR2^−^/^−^ co-infected (influenza-*Spn*) mice show reduced epithelial damage and are able to control *Spn* invasion (55). Alveolar macrophages (AMs) are resident phagocytic cells that provide the first line of defense against bacterial infections in the lungs (60). AMs are phagocytic, anti-inflammatory cells which maintain lung sterility and control pulmonary inflammation by preventing an influx of neutrophils during the early infection stage (61). An influenza infection promotes the apoptosis of AMs, leading to an increased permissiveness for *Spn* bacterial infections (43). The restoration of AMs in influenza-infected lungs confers resistance to *Spn* disease (62). Neutrophils are potent phagocytic cells with known anti-bacterial functions (63), and they massively accumulate in influenza and influenza-*Spn* co-infected mice. Prior reports suggest an impairment of neutrophil phagocytosis and intracellular reactive oxygen production in influenza-infected mice (64, 65), and neutrophil depletion was shown to be associated with an increased severity for *Spn* lung infection (64). However, dysregulated neutrophil recruitment is also associated with damage and pathology in infected tissues (66). While neutrophils have been shown to have a protective function in single *Spn* infections (67), their role in influenza-*Spn* co-infections remains unclear.

The levels of lung TNF-α elevate during a *Spn*-influenza co-infection, and its depletion exacerbates *Spn* infection (55). However, TNF-α responses are severely impaired during the early phase of co-infections (55), leading to a failure to control bacterial outgrowth and *Spn* infection. IL-6 limits influenza-induced inflammation and lung pathology as IL-6-deficient mice exhibited higher fibroblast accumulation, a lower extracellular matrix (ECM) turnover, and higher mortality (68). The critical role of IL-17 receptor (IL-17RA) signaling has been shown in acute immunopathology of influenza-infected lungs, as IL-17RA knockout mice had reduced tissue damage, reduced neutrophil numbers, and increased survival (69). Mucosal pre-exposure to Th17-inducing adjuvants have been shown to exacerbate pathology after an influenza infection (70), further establishing the role of IL-17 responses in influenza acute pathology. Therefore, an influenza infection promotes damaging inflammation in the airway, leading to permissiveness for *Spn* bacterial infections. A better understanding of the immune mediators implicated in influenza-mediated tissue pathology could lead to the development of therapeutic interventions to contain influenza pathology and subsequent *Spn* infections.

### Chronic inflammation and *Spn* infections

*Spn* causes significant morbidity and mortality worldwide in patients with chronic respiratory diseases, and vaccination is the most significant tool to contain *Spn* diseases among allergy and COPD patients (71). Several recent studies have shown a clear association between acute exacerbation and the isolation of bacterial species such as *Spn, Moraxella catarrhalis*, and *Haemophilus influenzae*, among the most frequently associated bacterial organisms (72). Consequently, Centers for Disease Control and Prevention (CDC) recommends the use of influenza and *Spn* vaccines (PCVs) in people with COPD and asthma conditions (71).

### Allergy, asthma airway pathology, and *Spn* infections

Asthma is a complex chronic inflammatory disease characterized by airway hyperresponsiveness, reversible airflow obstruction, and airway inflammation (73). The most common trigger for asthma is the continuous exposure to allergens, of which fungal agents are important factors (74, 75), and there is evidence for the presence of fungal sensitization in patients with asthma (76). Fungal asthma promotes a Th2-type response that mediates the production of cytokines such as IL-4, IL-5, IL-13, and IL-33 (77, 78), which leads to the recruitment of complex multi-factorial leukocyte eosinophils (79, 80). In murine models, Th2 cells play an important role in eosinophilic inflammation in fungal allergic asthma. Epithelial cell-derived cytokines such as thymic stromal lymphoprotein (TSLP), IL-25, and IL-33 promote eosinophilia by inducing IL-5 production (81). Additionally, dysregulation of the IL-17F/IL-17RC axis has been shown to predispose allergic inflammation in murine models of fungal allergic asthma (82). Besides murine models of fungal allergic asthma, mouse models using the house dust mite (HDM) also feature similarities to human allergic asthma, including eosinophilic lung inflammation and cytokines primarily associated with Th2-type inflammation (83). These animal models are used for evaluating the efficacy of anti-asthma drugs and exacerbations of bacterial/viral infections.

Allergic asthma consists of diverse immune phenotypes exhibiting differential lung pathology, remodeling of the respiratory tract, and mucociliary bronchial clearance (75, 84, 85). These confounding inflammatory immune mechanisms may promote or resist microbial infections in allergic human and animal infection models. Eosinophils are generally considered as major effector cells of asthma, and the eosinophilic response to viral infection has predominantly been shown to have a negative effect on human health (86). However, several studies have shown eosinophils may promote viral clearance and antiviral host defense. A recent study has shown that pulmonary eosinophilia linked with fungal allergic respiratory inflammation promotes antiviral host defenses against the influenza virus by promoting CD8^+^ T cell immunity (87). However, other studies have shown that viral infections exacerbate asthma and thereby promote bacterial infections (88). Since, influenza infections are an important determining factor for the development and exacerbation of *Spn* infections and a diverse spectrum of influenza strains cause infections in humans, animal models of influenza-*Spn* co-infections utilizing different influenza strains are required to study the role of asthma inflammation in influenza-dependent *Spn* diseases. The relationship between allergic airway inflammation and *Spn* pneumonia is not well understood. Recent studies report that asthma is associated with an increased risk of invasive *Spn* diseases (89, 90), and higher rates of *Spn* NP colonization, sinusitis, and otitis media have also been reported among individuals with asthma (91, 92). Additionally, *in vivo* model systems demonstrated that allergic airway inflammation was associated with a trend toward increased extrapulmonary *Spn* infections, highlighting the role of allergic airway inflammation in the development of invasive *Spn* diseases. Further studies that mimic diverse features of asthma phenotypes are needed to elucidate the precise mechanisms of the inflammatory response to *Spn* infections and the effectiveness of *Spn* vaccination in patients with asthma.

### COPD airway pathology and *Spn* infections

COPD is a heterogeneous entity that includes a variety of obstructive diseases that differ considerably on their mechanisms of action and responses to treatment. COPD is characterized by smoke-induced hypersecretion of mucus and emphysema in the pulmonary gas exchanging units (93). COPD and asthma are two distinct diseases with significantly different mechanisms of chronic inflammatory reactions (94). In COPD, the inflammation-associated changes are demonstrated predominantly in small airways and lung parenchyma, and result in tissue destruction with progressive, irreversible airflow restriction. The main changes in asthma are found in larger airways and may cause their intermittent and usually reversible obstruction (95, 96). Airway and alveolar epithelial cells play a central role in COPD inflammation (93). Epithelial cells are the primary source of proteolytic enzymes and chemoattractants responsible for the recruitment of immune effector cells implicated in the amplification and persistence of airway inflammation (93), and nitric oxide has been shown to be the most representative biomarker in exhaled air, originated from respiratory epithelium (97). Dendritic cells and macrophages are key innate immune effector cells in COPD inflammation (98). Activated macrophages and dendritic cells release inflammatory cytokines implicated in COPD pathology, such as TNF-α, IL-6, IL-8, IL-1β, and TGF-β (93, 98). Recent studies also implicate the pathogenic role of IL-17, since IL-17RA-deficient mice were protected from airway inflammation and fibrosis in COPD-like models (99). Additionally, lymphocytes and neutrophil recruitment in the inflamed airway/lungs further promote airway damage (61). The persistent inflammation in COPD allows for pathological airway and vascular remodeling, which involves the deposition of an extracellular matrix in sub-epithelial layers and hypertrophy of smooth muscles, leading to the thickening of airway walls and the narrowing of bronchial lumen. Therefore, COPD-mediated pulmonary pathology compromises tissue barrier integrity, which leads to an increased permissiveness for bacterial infections (100).

*Spn* plays an important role in causing acute exacerbations in patients with chronic respiratory diseases (101). A number of *Spn* serotypes have shown to be associated with colonization and exacerbations in COPD patients (72, 101–103). Currently available *Spn* vaccines could cover almost 50% of the *Spn* serotypes in COPD patients (72). However, due to the evolution of non-vaccine *Spn* serotypes and the limitation of currently available *Spn* vaccines, understanding the role of COPD inflammation in the development of *Spn* diseases is central to designing an intervention to prevent *Spn* colonization and diseases. Pulmonary inflammation, mucociliary dysfunction, and airway remodeling are central components of COPD-mediated immune pathology, implicated in the permissiveness of airway infections (104). The tracheobronchial mucilliary movement is central to maintaining the sterility of the lower respiratory tract by transporting bacteria trapped in mucus toward the pharynx. COPD causes mucociliary dysfunction, thus results in the promotion of persistent airway bacterial colonization (105, 106). Therefore, anti-inflammatory therapies intended to improve mucociliary clearance have the potential to modulate a pulmonary defense against microbial pathogens.

## Conclusion

Despite currently available vaccines, *Spn* continues to colonize human populations, and *Spn* carriage thus represents a significant medical problem. Airway tissue damage and hyper-inflammation is one of the most significant risk factors for the transition of *Spn* carriage into a disease. An acute or chronic inflammatory condition caused by airway viral infection or conditions such as COPD and asthma are major risk factors for the manifestation of *Spn* diseases, and CDC recommends the use of seasonal influenza and *Spn* vaccines in these human populations. A carefully orchestrated inflammatory response is required for the resolution of airway bacterial infections, and defects in the regulation of inflammation that arise from respiratory conditions become a contributor to tissue pathology, thus leading to an increased permissiveness for *Spn* bacterial infections. Traditionally, a majority of murine infection models employed in the investigating of *Spn* diseases typically used *Spn* single infection conditions. These mice models used infection inocula in low or high volumes intended to develop colonization or a pneumonia infection. However, a significant shift has occurred with the development of mouse infection models mimicking the natural infections of *Spn* bacteria. Influenza-*Spn* co-infection models are increasingly being used to study the role of viral-induced acute airway inflammation in *Spn* infections. Similarly, mouse models of chronic inflammation such as allergy or COPD provide a platform to study *Spn* infections in conditions similar to natural bacterial infections. Therefore, the utilization of risk factors in murine infection models will lead to a better understanding of the dynamics of microbial interplay and the inflammatory mechanisms implicated in *Spn* infections. The knowledge gained could be harnessed in the design of novel therapeutic interventions to circumvent *Spn* bacterial infections in at-risk human populations.

## Author contributions

MK conceived the idea, drafted the outline of the article, and supervised through all stages of its preparation. IS collected the review literature and participated in the writing of first draft. SG, SM and SZ participated in the writing of first draft.

### Conflict of interest statement

The authors declare that the research was conducted in the absence of any commercial or financial relationships that could be construed as a potential conflict of interest.
